# PARP-1 activity is required for the reconsolidation and extinction of contextual fear memory

**DOI:** 10.1186/s13041-015-0153-7

**Published:** 2015-10-15

**Authors:** Hiroyoshi Inaba, Akinori Tsukagoshi, Satoshi Kida

**Affiliations:** Department of Bioscience, Faculty of Applied Bioscience, Tokyo University of Agriculture, Tokyo, 156-8502 Japan; Core Research for Evolutional Science and Technology, Japan Science and Technology Agency, Saitama, 332-0012 Japan

**Keywords:** Poly (ADP-ribose) polymerase, Poly ADP-ribosylation, Hippocampus, Medial prefrontal cortex, Fear conditioning, 3-aminobenzamide, PJ34, Tiq-A, Gene expression, c-fos

## Abstract

**Background:**

Memory consolidation, reconsolidation, and extinction have been shown to require new gene expression. Poly ADP-ribosylation mediated by poly (ADP-ribose) polymerase-1 (PARP-1) is known to regulate transcription through histone modification. Recent studies have suggested that PARP-1 positively regulates the formation of long-term memory (LTM); however, the roles of PARP-1 in memory processes, especially processes after retrieval, remain unknown.

**Results:**

Here, we show critical roles for PARP-1 in the consolidation, reconsolidation, and extinction of contextual fear memory in mice. We examined the effects of pharmacological inhibition of PARP-1 activity in the hippocampus or medial prefrontal cortex (mPFC) on these memory processes. Similarly with previous findings, a micro-infusion of the PARP-1 inhibitor 3-aminobenzamide or PJ34 into the dorsal hippocampus, but not mPFC, impaired LTM formation without affecting short-term memory (STM). Importantly, this pharmacological blockade of PARP-1 in the dorsal hippocampus, but not mPFC, also disrupted post-reactivation LTM without affecting post-reactivation STM. Conversely, micro-infusion of the PARP-1 inhibitors into the mPFC, but not dorsal hippocampus, blocked long-term extinction. Additionally, systemic administration of the PARP-1 inhibitor Tiq-A blocked c-fos induction in the hippocampus, which is observed when memory is consolidated or reconsolidated, and also blocked c-fos induction in the mPFC, which is observed when memory is extinguished.

**Conclusions:**

Our observations showed that PARP-1 activation is required for the consolidation, reconsolidation, and extinction of contextual fear memory and suggested that PARP-1 contributes to the new gene expression necessary for these memory processes.

## Background

Poly ADP-ribosylation of proteins is one of the reversible post-translational modifications in eukaryotes and, importantly, is involved in biological processes in the nucleus such as transcriptional regulation, DNA repair, and control of centrosomal division [1–3]. This poly ADP-ribosylation is mainly catalyzed by the nuclear protein poly (ADP-ribose) polymerase-1 (PARP-1) [4–6]. Recent studies have shown that PARP-1 is expressed in the central nervous system and is activated by neural activity such as neuronal depolarization and stimulation by nerve growth factor [7–9].

Short-term memory (STM) is labile. To generate a stable long-term memory (LTM), a memory is stabilized through a process known as memory consolidation [10–12]. Previous studies have revealed that when an LTM is retrieved, the memory returns to the labile state again and is re-stabilized through the process of reconsolidation, which is similar to that of consolidation [13–16]. Conversely, memory extinction is induced by the continuous or repeated retrieval of a fear memory, thereby inhibiting fear responses [17–20]. The most common and critical biochemical step of memory consolidation, reconsolidation, and extinction is the requirement of new gene expression [11, 13, 21–25]. Importantly, there is increasing evidence that post-translational modifications, such as phosphorylation, acetylation, methylation, ubiquitination, and SUMOylation, which regulate gene expression, play an important role in the formation of LTM [26–35].

A contextual fear memory is an associative memory of a context with conditioned fear arising from a stimulus or event, such as an electrical footshock. Memory consolidation and reconsolidation of contextual fear have been shown to depend on the hippocampus and require gene expression in this brain region [23, 36–41]. In contrast, the medial prefrontal cortex (mPFC) is important for long-term fear extinction, which also requires gene expression in this brain region [24, 39, 42–44].

Recent pharmacological studies have shown that PARP-1-mediated poly ADP-ribosylation of nuclear proteins in *Aplysia* is necessary for gene expression-dependent long-term facilitation and memory formation [45, 46]. Likewise, there are reports that PARP-1 activation in rodents is required for long-term potentiation and hippocampus-dependent memory consolidation [47–50]. However, the role of PARP-1 in gene expression-dependent memory processes such as memory reconsolidation and extinction remains unclear. In the present study, we clarified the role of PARP-1 in the regulation of contextual fear memory. To do this, we analyzed the effects of the pharmacological inhibition of PARP-1 activity in the hippocampus and mPFC on memory consolidation, reconsolidation, and extinction of contextual fear.

## Results

### PARP-1 activity in the hippocampus is required for the consolidation of contextual fear memory

**Fig. 1:**
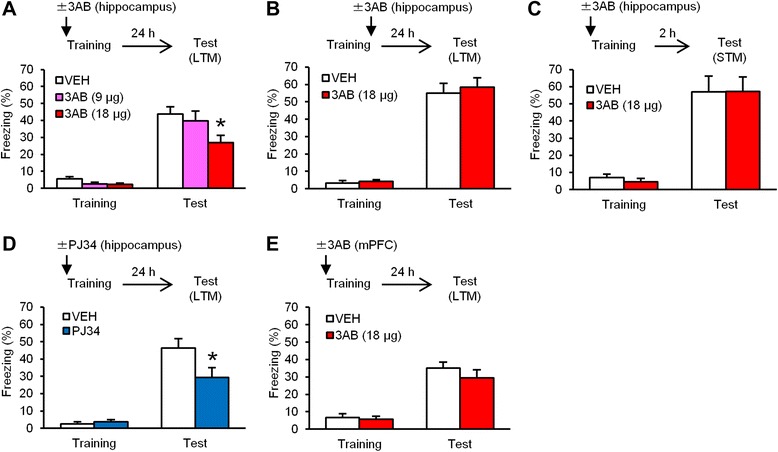
Inhibition of PARP-1 activity in the hippocampus blocks the consolidation of contextual fear memory. **a** Effects of a micro-infusion of low- or high-dose of 3AB into the dorsal hippocampus at 5 min before training on LTM (VEH, *n* = 12; 3AB-9 μg, *n* = 12; 3AB-18 μg, *n* = 12). **b** Effects of a micro-infusion of 3AB into the dorsal hippocampus immediately after training on LTM (VEH, *n* = 13; 3AB, *n* = 14). **c** Effects of a micro-infusion of 3AB into the dorsal hippocampus at 5 min before training on STM (VEH, *n* = 8; 3AB, *n* = 8). **d** Effects of a micro-infusion of PJ34 into the dorsal hippocampus at 5 min before training on LTM (VEH, *n* = 9; PJ34, *n* = 10). **e** Effects of a micro-infusion of 3AB into the mPFC at 5 min before training on LTM (VEH, *n* =15; 3AB, *n* = 15). *p < 0.05, compared with the VEH group at the test. Error bars indicate SEM

Abundant studies have shown that the consolidation of contextual fear memory depends on the hippocampus [36–38]. Importantly, recent studies using pre-training administration of PARP-1 inhibitors into the dorsal hippocampus or lateral ventricle have shown that inhibition of PARP-1 activity blocks memory consolidation [47–49]. To understand further the roles of PARP-1 in memory consolidation, we first asked whether hippocampal PARP-1 activity is required for the consolidation of hippocampus-dependent contextual fear memory in mice. To do this, the mice were trained with a single footshock and tested 24 h later. They received a micro-infusion of the PARP-1 inhibitor 3-aminobenzamide (3AB; low- [9 μg/side] or high-dose [18 μg/side]) or vehicle (VEH) into the dorsal hippocampus at 5 min before (Fig. 1a) or immediately after (Fig. 1b) training. One-way analysis of variance (ANOVA) revealed a significant effect of drug when the mice received 3AB before, but not immediately after, training (pre-training infusion: *F*_(2,33)_ = 3.419, *p* < 0.05; post-training infusion: *F*_(1,25)_ = 0.176, *p* > 0.05; Fig. 1a and b). *Post hoc* Newman-Keuls analysis revealed that mice treated with 3AB pre-training froze significantly less than the VEH group in a dose-dependent manner (VEH vs. low-dose: *p* > 0.05; VEH vs. high-dose: *p* < 0.05; Fig. 1a). In contrast, mice treated with 3AB post-training showed comparable freezing with the VEH group (*p* > 0.05; Fig. 1b). These observations indicated that pre-, but not post-, training inhibition of hippocampal PARP-1 impairs LTM formation of contextual fear.

We next examined the effect of a pre-training infusion of 3AB on STM (2 h memory). We performed a similar experiment as in Fig. 1a, except the mice were treated with 3AB (18 μg/side) at 5 min before training and tested at 2 h after training. One-way ANOVA revealed no significant effect of drug (*F*_(1,14)_ = 0.0003, *p* > 0.05; Fig. 1c). This observation indicated that the inhibition of hippocampal PARP-1 activity does not affect STM.

We examined the effects of another PARP-1 inhibitor (PJ34) on LTM. We performed a similar experiment as in Fig. 1a, except the mice received a micro-infusion of PJ34 at 5 min before training. One-way ANOVA revealed a significant effect of drug (*F*_(1,17)_ = 4.639, *p* < 0.05; Fig. 1d). The PJ34 group froze significantly less than the VEH group (*p* < 0.05; Fig. 1d). These results were consistent with our observation in Fig. 1a and indicated that inhibition of hippocampal PARP-1 blocks LTM formation of contextual fear.

As a control experiment, we examined the effects of inhibiting PARP-1 activity in the mPFC on LTM formation of contextual fear. We performed a similar experiment as in Fig. 1a, except the mice received a micro-infusion of 3AB (18 μg) into the mPFC at 5 min before training. One-way ANOVA revealed no significant effect of drug (*F*_(1,28)_ = 1.037, *p* > 0.05; Fig. 1e). This observation suggested that PARP-1 activity in the mPFC is not required for the formation of contextual fear memory.

Taken together, similarly to previous findings [47–49], we observed that inhibition of PARP-1 activity in the hippocampus impairs LTM formation of contextual fear without affecting STM. Our results suggested that hippocampal PARP-1 activity is required for the consolidation of contextual fear memory.

### PARP-1 activity in the hippocampus is required for the reconsolidation of contextual fear memory

**Fig. 2:**
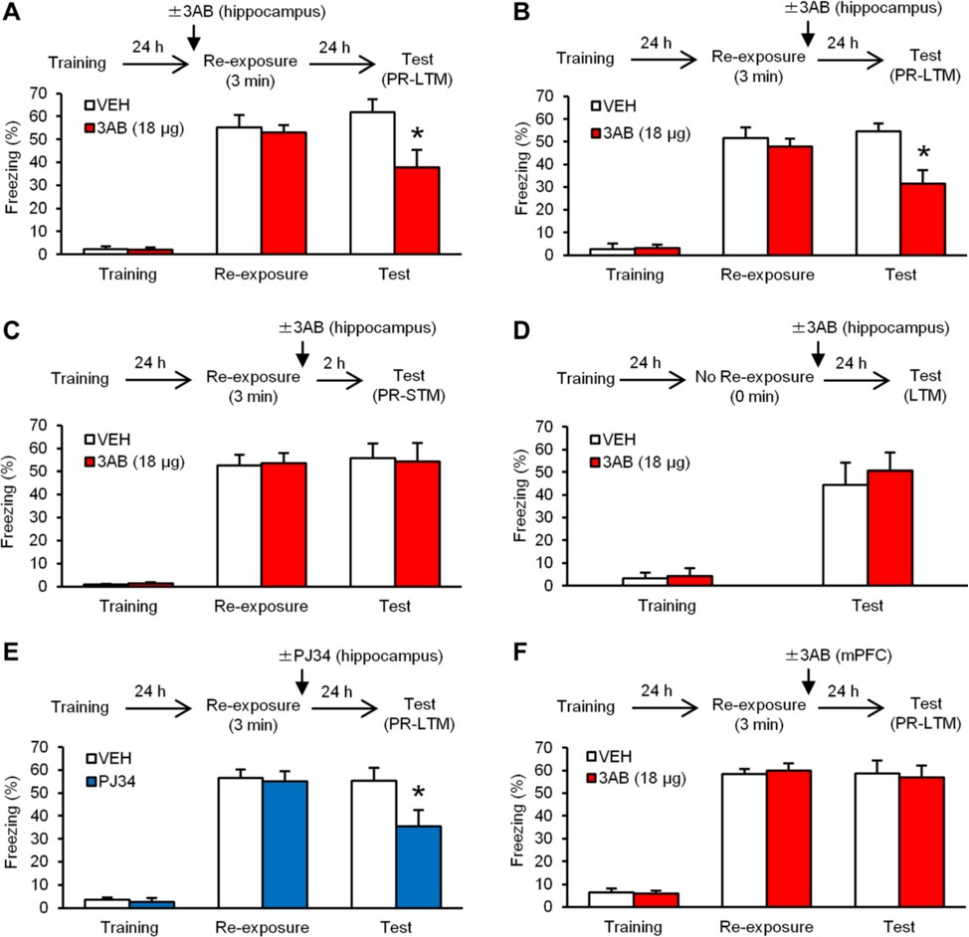
Inhibition of PARP-1 activity in the hippocampus blocks the reconsolidation of contextual fear memory. **a**, **b** Effects of a micro-infusion of 3AB into the dorsal hippocampus at 5 min before (**a**) or immediately after (**b**) the 3 min re-exposure on PR-LTM (pre-re-exposure infusion: VEH, *n* = 9; 3AB, *n* = 10; post-re-exposure infusion: VEH, *n* = 9; 3AB, *n* = 10). **c** Effects of a micro-infusion of 3AB into the dorsal hippocampus immediately after the 3 min re-exposure on PR-STM (VEH, *n* = 10; 3AB, *n* = 8). **d** Effects of a micro-infusion of 3AB into the dorsal hippocampus at 24 h after training without re-exposure on LTM (VEH, *n* = 9; 3AB, *n* = 10). **e** Effects of a micro-infusion of PJ34 into the dorsal hippocampus immediately after the 3 min re-exposure on PR-LTM (VEH, *n* = 7; PJ34, *n* = 7). **f** Effects of a micro-infusion of 3AB into the mPFC immediately after the 3 min re-exposure on PR-LTM (VEH, *n* = 9; 3AB, *n* = 10). *p < 0.05, compared with the VEH group at the test. Error bars indicate SEM

Reconsolidation has been shown to involve molecular processes similar to those of consolidation [13–16, 23, 39]. Furthermore, similarly to consolidation, the reconsolidation of contextual memory depends on new gene expression in the hippocampus [23, 39–41]. Therefore, it is possible that hippocampal PARP-1 activity is required for the reconsolidation of contextual fear memory. To assess this, we examined the effects of inhibiting hippocampal PARP-1 on post-reactivation LTM (PR-LTM) of contextual fear [25]. The mice were trained and 24 h later, they were re-exposed to the training context for 3 min (re-exposure). PR-LTM was tested at 24 h after re-exposure (test). The mice received a micro-infusion of 3AB (18 μg/side) or VEH into the dorsal hippocampus at 5 min before (Fig. 2a) or immediately after (Fig. 2b) re-exposure. During re-exposure, comparable levels of freezing were observed between the VEH and 3AB groups (pre-re-exposure infusion: *F*_(1,17)_ = 0.112, *p* > 0.05; post-re-exposure infusion: *F*_(1,17)_ = 0.429, *p* > 0.05; Fig. 2a and b). This observation indicated that inhibiting PARP-1 activity does not affect the retrieval of contextual fear memory. Two-way ANOVA revealed significant drug (VEH vs. 3AB) × time (re-exposure vs. test) interactions (pre-re-exposure infusion: drug, *F*_(1,17)_ = 5.140, *p* > 0.05 ; time, *F*_(1,17)_ = 1.285, *p* > 0.05; interaction, *F*_(1,17)_ = 8.333, *p* < 0.05; post-re-exposure infusion: drug, *F*_(1,17)_ = 5.938, *p* < 0.05; time, *F*_(1,17)_ = 3.633, *p* > 0.05; interaction, *F*_(1,17)_ = 7.505, *p* < 0.05; Fig. 2a and b). *Post hoc* Newman-Keuls analysis revealed that the 3AB groups froze significantly less than the VEH groups during the test (pre-re-exposure infusion: *p* < 0.05; post-re-exposure infusion: *p* < 0.05; Fig. 2a and b). These results indicated that in contrast to the results of the consolidation experiments (Fig. 1a and b), post- as well as pre-re-exposure inhibition of PARP-1 activity in the hippocampus disrupted PR-LTM, suggesting that the reconsolidation of contextual fear memory shows a similar requirement for PARP-1 activation as consolidation, but has different time windows for this activation.

We next examined the effect of 3AB infusion on post-reactivation STM (PR-STM). The mice received a micro-infusion of 3AB immediately after re-exposure and 2 h later, they were tested. Two-way ANOVA revealed no significant effect of drug and time or a drug × time interaction (drug, *F*_(1,16)_ = 0.001, *p* > 0.05; time, *F*_(1,16)_ = 0.127, *p* > 0.05; interaction, *F*_(1,16)_ = 0.054, *p* > 0.05; Fig. 2c). This observation indicated that inhibiting PARP-1 activity in the hippocampus does not affect PR-STM.

As a control experiment, we asked whether the disruption of contextual fear memory by 3AB depends on memory retrieval. We performed a similar experiment as in Fig. 2b, except the mice were not re-exposed to the context. One-way ANOVA revealed no significant effect of drug (*F*_(1,17)_ = 0.237, *p* > 0.05; Fig. 2d). This result indicated that inhibiting PARP-1 activity failed to disrupt contextual fear memory when the memory was not retrieved. Taken together, these results suggested that inhibiting hippocampal PARP-1 activity impairs the reconsolidation of contextual fear memory following retrieval.

We observed similar results using a micro-infusion of PJ34. We performed a similar experiment as in Fig. 2b, except the mice received a micro-infusion of PJ34 immediately after re-exposure. Two-way ANOVA revealed a significant drug × time interaction (drug, *F*_(1,12)_ = 2.773, *p* > 0.05; time, *F*_(1,12)_ = 6.410, *p* < 0.05; interaction, *F*_(1,12)_ = 4.929, *p* < 0.05; Fig. 2e). The PJ34 group froze significantly less than the VEH group during the test (*p* < 0.05; Fig. 2e). These results support our finding in Fig. 2b and indicated that inhibiting hippocampal PARP-1 activity blocks memory reconsolidation.

Finally, we examined the effects of inhibiting PARP-1 activity in the mPFC on the reconsolidation of contextual fear memory. We performed a similar experiment as in Fig. 2b, except the mice received a micro-infusion of 3AB (18 μg) into the mPFC immediately after re-exposure. Two-way ANOVA revealed no significant effects of drug and time or a drug × time interaction (drug, *F*_(1,17)_ = 0.0003, *p* > 0.05; time, *F*_(1,17)_ = 0.170, *p* > 0.05; interaction, *F*_(1,17)_ = 0.202, *p* > 0.05; Fig. 2f). This observation indicated that inhibiting PARP-1 activity in the mPFC is not required for the reconsolidation of contextual fear memory.

Taken together, these observations that inhibiting PARP-1 activity in the hippocampus, but not mPFC, disrupts PR-LTM without affecting PR-STM, suggested that hippocampal PARP-1 activity is required for the reconsolidation of contextual fear memory.

### PARP-1 activity in the mPFC is required for the long-term extinction of contextual fear memory

**Fig. 3:**
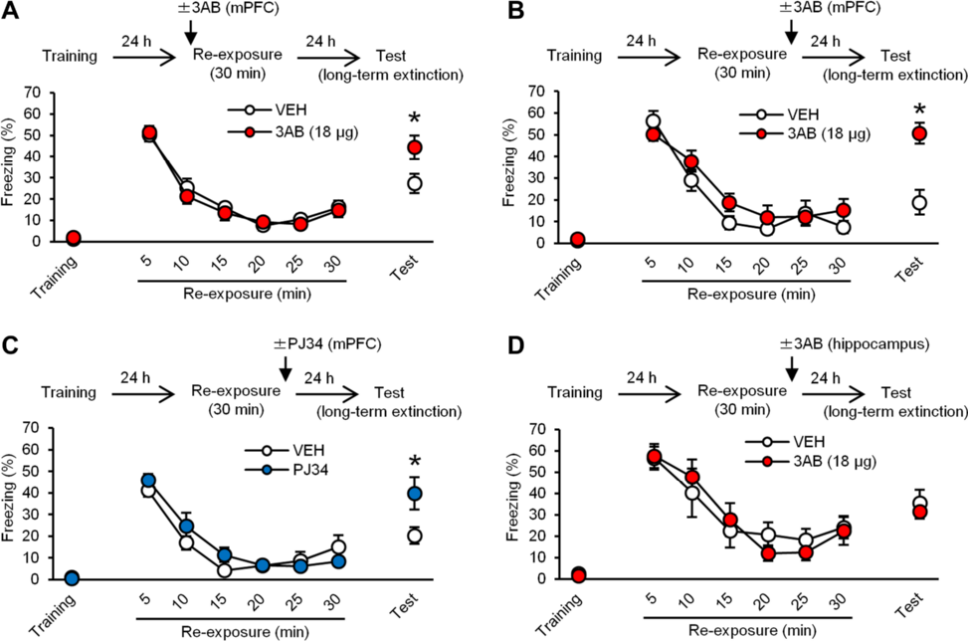
Inhibition of PARP-1 activity in the mPFC blocks the long-term extinction of contextual fear memory. **a**, **b** Effects of a micro-infusion of 3AB into the mPFC at 5 min before (**a**) or immediately after (**b**) the 30 min re-exposure on long-term extinction (pre-re-exposure infusion: VEH, *n* = 10; 3AB, *n* = 11; post-re-exposure infusion: VEH, *n* = 7; 3AB, *n* = 7). **c** Effects of a micro-infusion of PJ34 into the mPFC immediately after the 30 min re-exposure on long-term extinction (VEH, *n* = 8; PJ34, *n* = 7). **d** Effects of a micro-infusion of 3AB into the dorsal hippocampus immediately after the 30 min re-exposure on long-term extinction (VEH, *n* = 9; 3AB, *n* = 9). *p < 0.05, compared with the VEH group at the test. Error bars indicate SEM

Previous studies have shown that not only consolidation and reconsolidation but also the long-term extinction of fear memory depend on gene expression, suggesting that long-term extinction shows similar molecular signatures as consolidation and reconsolidation [24, 25, 39]. However, a previous study showed that the long-term extinction of contextual fear memory depends on the mPFC, but not hippocampus [39]. Therefore, we examined the effects of inhibiting PARP-1 activity in the mPFC on the extinction of contextual fear memory [39]. The mice were trained, and 24 h later, they were re-exposed to the context for 30 min. Extinction memory was tested at 24 h after re-exposure. The mice received a micro-infusion of 3AB (18 μg) or VEH into the mPFC at 5 min before (Fig. 3a) or immediately after (Fig. 3b) re-exposure. The VEH and 3AB groups showed that freezing levels decreased over time with re-exposure (pre-re-exposure infusion: *F*_(5,95)_ = 68.119, *p* < 0.05; post-re-exposure infusion: *F*_(5,60)_ = 58.017, *p* < 0.05; Fig. 3a and b), and that overall freezing levels did not differ during re-exposure (pre-re-exposure infusion: *F*_(1,19)_ = 0.241, *p* > 0.05; post-re-exposure infusion: *F*_(1,12)_ = 0.769, *p* > 0.05; Fig. 3a and b). These results indicated that both groups displayed comparable within-session extinction, suggesting that inhibiting PARP-1 activity in the mPFC does not affect within-session extinction. Two-way ANOVA revealed a significant drug × time (last 5 min in re-exposure vs. test) interaction (pre-re-exposure infusion: drug, *F*_(1,19)_ = 3.083, *p* > 0.05; time, *F*_(1,19)_ = 25.892, *p* < 0.05; interaction, *F*_(1,19)_ = 5.291, *p* < 0.05; post-re-exposure infusion: drug, *F*_(1,12)_ = 16.056, *p* < 0.05; time, *F*_(1,12)_ = 27.813, *p* < 0.05; interaction, *F*_(1,12)_ = 7.458, *p* < 0.05; Fig. 3a and b). *Post hoc* Newman-Keuls analysis revealed that the 3AB groups froze significantly more than the VEH groups during the test (pre-re-exposure infusion: *p* < 0.05; post-re-exposure infusion: *p* < 0.05; Fig. 3a and b). Importantly, measurements at 24 h after this 30 min re-exposure confirmed that the extinction of conditioned freezing persisted in the VEH groups (first 5 min in re-exposure vs. test; pre-re-exposure infusion: *t*_(9)_ = 4.276, *p* < 0.05; post-re-exposure infusion: *t*_(6)_ = 6.267, *p* < 0.05; Fig. 3a and b), indicating that the VEH groups showed long-term extinction. These results indicated that inhibiting PARP-1 activity in the mPFC blocks long-term extinction, suggesting that PARP-1 in the mPFC is required for this process.

We observed similar results using a micro-infusion of PJ34. The mice were treated similarly as above, except they received a micro-infusion of PJ34 immediately after re-exposure. The VEH and PJ34 groups showed comparable within-session extinction (decrease over time, *F*_(5,65)_ = 42.074, *p* < 0.05; overall freezing, *F*_(1,13)_ = 0.433, *p* > 0.05; Fig. 3c). Two-way ANOVA revealed a significant drug × time interaction (drug, *F*_(1,13)_ = 1.462, *p* > 0.05; time, *F*_(1,13)_ = 14.789, *p* < 0.05; interaction, *F*_(1,13)_ = 7.510, *p* < 0.05; Fig. 3c). The PJ34 group froze significantly more than the VEH group during the test (*p* < 0.05; Fig. 3c). These results indicated that inhibiting PARP-1 activity in the mPFC with PJ34 blocks long-term extinction.

Finally, we examined the effects of inhibiting hippocampal PARP-1 activity on long-term extinction. We performed a similar experiment as in Fig. 3b, except the mice received a micro-infusion of 3AB (18 μg/side) into the dorsal hippocampus. The VEH and 3AB groups showed comparable within-session extinction (decrease over time, *F*_(5,80)_ = 21.435, *p* < 0.05; overall freezing; *F*_(1,16)_ = 0.004, *p* > 0.05; Fig. 3d). In contrast to the result of the mPFC, two-way ANOVA revealed no significant effects of drug and time or a drug × time interaction (drug, *F*_(1,16)_ = 0.243, *p* > 0.05; time, *F*_(1,16)_ = 4.085, *p* > 0.05; interaction, *F*_(1,16)_ = 0.052, *p* > 0.05; Fig. 3d). These results indicated that PARP-1 activity in the hippocampus is not required for long-term extinction.

Taken together, our observations suggested that PARP-1 activity in the mPFC, but not hippocampus, is required for the long-term extinction of contextual fear memory.

### PARP-1 activity is required for gene expression during the consolidation, reconsolidation, and extinction of contextual fear memory

The consolidation, reconsolidation, and extinction of contextual fear memory are known to depend on new gene expression [23, 25, 36, 39]. It is possible that PARP-1 contributes to these memory processes through the activation of gene expression. To assess this possibility, we examined the effects of PARP-1 inactivation on the expression of the immediate-early gene c-fos, which is induced in these memory processes [24, 39, 51–53].

#### Consolidation

**Fig. 4:**
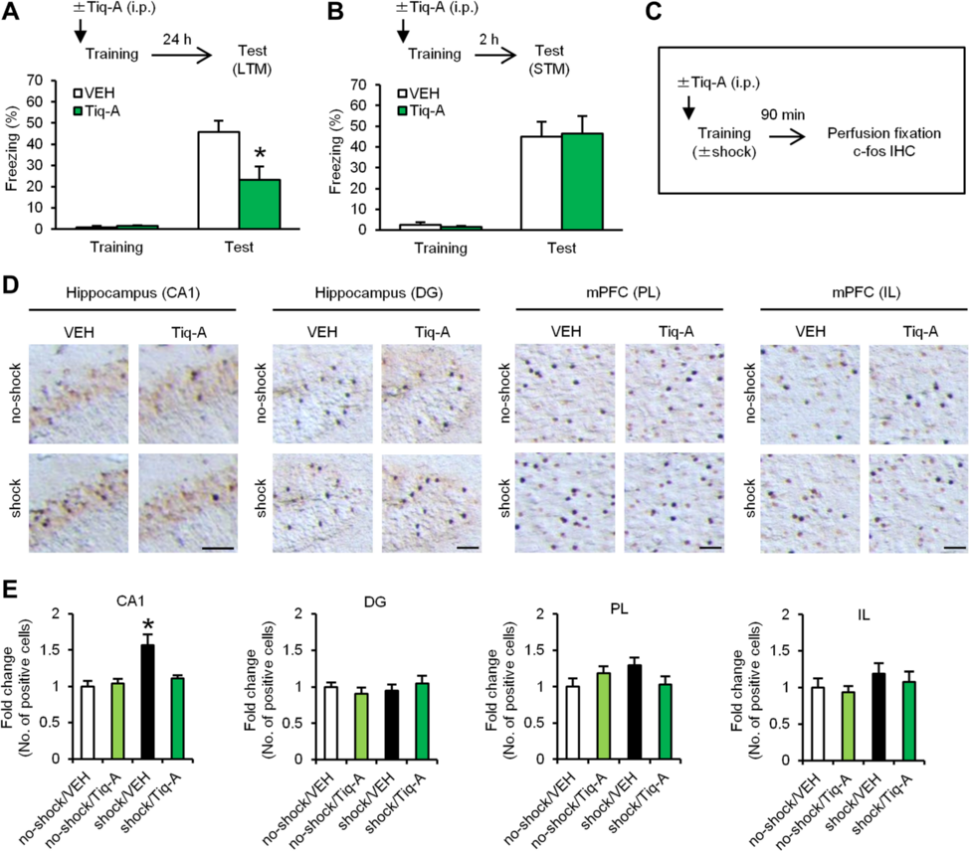
PARP-1 inactivation blocks c-fos induction in the hippocampal CA1 region when memory is consolidated. **a**, **b** Effects of a systemic injection of Tiq-A at 30 min before training on LTM (**a**) and STM (**b**) (LTM: VEH, *n* = 8; Tiq-A, *n* = 8; STM: VEH, *n* = 10; Tiq-A, *n* = 10). *p < 0.05, compared with the VEH group at the test. **c** Experimental design for IHC. **d** Representative immunohistochemical staining of c-fos-positive cells in the CA1, DG, PL, and IL regions from the indicated mice. Scale bar, 50 μm. **e** Expression of c-fos in the CA1, DG, PL, and IL regions (*n* = 7–8 for each group). The expression of c-fos in each group is expressed as the ratio of the no-shock/VEH group to the other groups. *p < 0.05, compared with the other groups. Error bars indicate SEM

We first examined the effects of a systemic injection of the brain-permeable PARP-1 inhibitor Tiq-A on the consolidation of contextual fear memory at the behavioral level. We performed similar experiments as in Fig. 1a and c, except the mice were systemically injected with Tiq-A at 30 min before training. One-way ANOVA revealed a significant effect of drug when the mice were tested at 24 h, but not at 2 h, after training (24 h memory: *F*_(1,14)_ = 6.424, *p* < 0.05; 2 h memory: *F*_(1,18)_ = 0.036, *p* > 0.05; Fig. 4a and b). The Tiq-A group froze significantly less than the VEH group at 24 h after training (*p* < 0.05; Fig. 4a). Similarly to Fig. 1, these observations indicated that PARP-1 inhibition by Tiq-A blocks the consolidation of contextual fear memory.

We measured the number of c-fos-positive cells in the hippocampus and mPFC at 90 min after training using immunohistochemistry (IHC). Two groups were trained with a footshock (shock groups), and the remaining two groups did not receive a footshock (no-shock groups). At 30 min before training, these groups were systemically injected with Tiq-A or VEH (shock/Tiq-A, shock/VEH, no-shock/Tiq-A, and no-shock/VEH groups) (Fig. 4c). Two-way ANOVA revealed a significant conditioning × drug interaction only in the CA1 region of the hippocampus (conditioning, *F*_(1,27)_ = 13.273, *p* < 0.05; drug, *F*_(1,27)_ = 5.532, *p* < 0.05; interaction, *F*_(1,27)_ = 7.742, *p* < 0.05; Fig. 4d and e), but not in the dentate gyrus (DG) region of the hippocampus or prelimbic (PL) and infralimbic (IL) regions of the mPFC (DG: conditioning, *F*_(1,27)_ = 0.237, *p* > 0.05; drug, *F*_(1,27)_ = 0.000, *p* > 0.05; interaction, *F*_(1,27)_ = 1.201, *p* > 0.05; PL: conditioning, *F*_(1,27)_ = 0.411, *p* > 0.05; drug, *F*_(1,27)_ = 0.117, *p* > 0.05; interaction, *F*_(1,27)_ = 4.180, *p* > 0.05; IL: conditioning, *F*_(1,27)_ = 1.713, *p* > 0.05; drug, *F*_(1,27)_ = 0.491, *p* > 0.05; interaction, *F*_(1,27)_ = 0.039, *p* > 0.05; Fig. 4d and e). The shock/VEH group showed significantly more c-fos-positive cells in the hippocampal CA1 area compared with the other groups (*p* < 0.05; Fig. 4d and e). These results indicated that PARP-1 inactivation by Tiq-A blocks c-fos induction in the hippocampus when memory is consolidated.

#### Reconsolidation

**Fig. 5:**
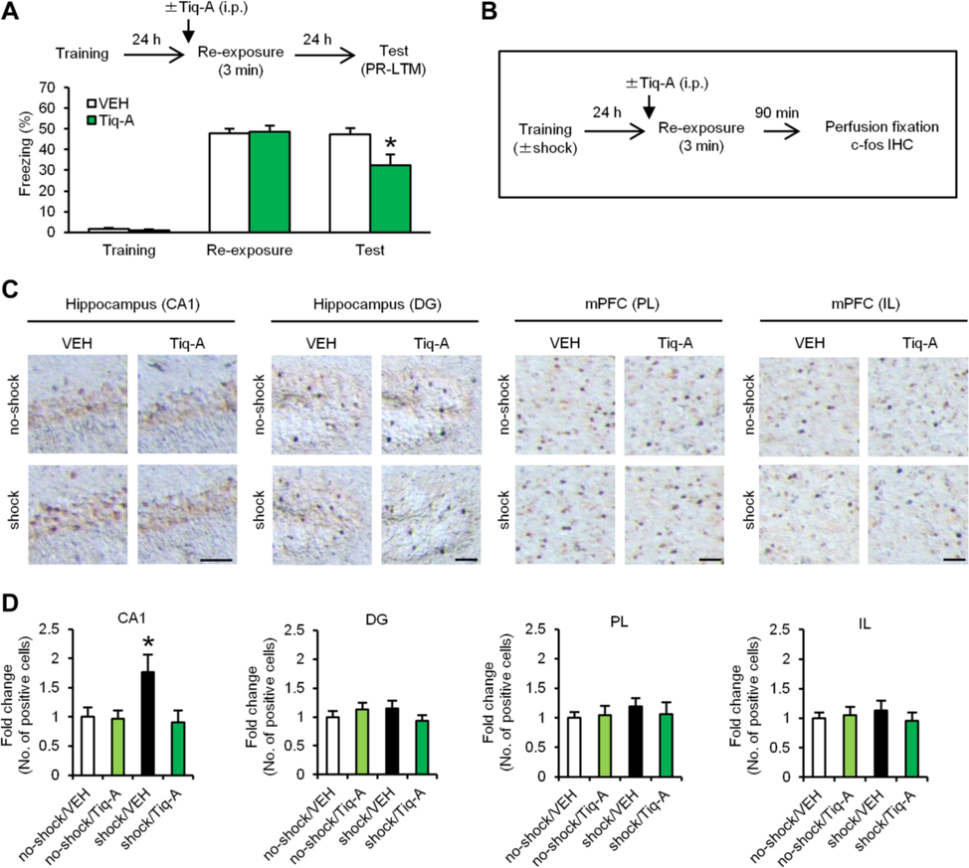
PARP-1 inactivation blocks c-fos induction in the hippocampal CA1 region when memory is reconsolidated. **a** Effects of the systemic injection of Tiq-A at 30 min before the 3 min re-exposure on PR-LTM (VEH, *n* = 10; Tiq-A, *n* = 10). *p < 0.05, compared with the VEH group at the test. **b** Experimental design for IHC. **c** Representative immunohistochemical staining of c-fos-positive cells in the CA1, DG, PL, and IL regions from the indicated mice. Scale bar, 50 μm. **d** Expression of c-fos in the CA1, DG, PL, and IL regions (*n* = 11–17 for each group). The expression of c-fos in each group is expressed as the ratio of the no-shock/VEH group to the other groups. *p < 0.05, compared with the other groups. Error bars indicate SEM

To examine whether the systemic injection of Tiq-A blocks the reconsolidation of contextual fear memory, we performed a similar experiment as in Fig. 2a, except the mice were systemically injected with Tiq-A at 30 min before the 3 min re-exposure. During re-exposure, comparable levels of freezing were observed between the VEH and Tiq-A groups (*F*_(1,18)_ = 0.035, *p* > 0.05; Fig. 5a). Two-way ANOVA revealed a significant drug × time (re-exposure vs. test) interaction (drug, *F*_(1,18)_ = 3.082, *p* > 0.05 ; time, *F*_(1,18)_ = 8.143, *p* < 0.05; interaction, *F*_(1,18)_ = 7.559, *p* < 0.05; Fig. 5a). The Tiq-A group froze significantly less than the VEH group during the test (*p* < 0.05; Fig. 5a). Similarly to Fig. 2, these observations indicated that PARP-1 inactivation by Tiq-A blocks the reconsolidation of contextual fear memory.

Similarly to Fig. 4, we performed IHC at 90 min after the 3 min re-exposure using four groups (shock/Tiq-A, shock/VEH, no-shock/Tiq-A, and no-shock/VEH groups). At 30 min before re-exposure, these groups were systemically injected with Tiq-A or VEH (Fig. 5b). Two-way ANOVA revealed a significant conditioning × drug interaction in the hippocampal CA1 region (conditioning, *F*_(1,52)_ = 3.215, *p* > 0.05; drug, *F*_(1,52)_ = 5.169, *p* < 0.05; interaction, *F*_(1,52)_ = 4.420, *p* < 0.05; 5c and d), but not in the DG, PL, and IL regions (DG: conditioning, *F*_(1,52)_ = 0.052, *p* > 0.05; drug, *F*_(1,52)_ = 0.106, *p* > 0.05; interaction, *F*_(1,52)_ = 2.130, *p* > 0.05; PL: conditioning, *F*_(1,52)_ = 0.490, *p* > 0.05; drug, *F*_(1,52)_ = 0.074, *p* > 0.05; interaction, *F*_(1,52)_ = 0.363, *p* > 0.05; IL: conditioning, *F*_(1,52)_ = 0.024, *p* > 0.05; drug, *F*_(1,52)_ = 0.208, *p* > 0.05; interaction, *F*_(1,52)_ = 0.649, *p* > 0.05; Fig. 5c and d). The shock/VEH group showed significantly more c-fos-positive cells in the CA1 area compared with the other groups (*p* < 0.05; Fig. 5c and d). These results indicated that PARP-1 inactivation blocks c-fos induction in the hippocampus when memory is reconsolidated.

#### Extinction

**Fig. 6:**
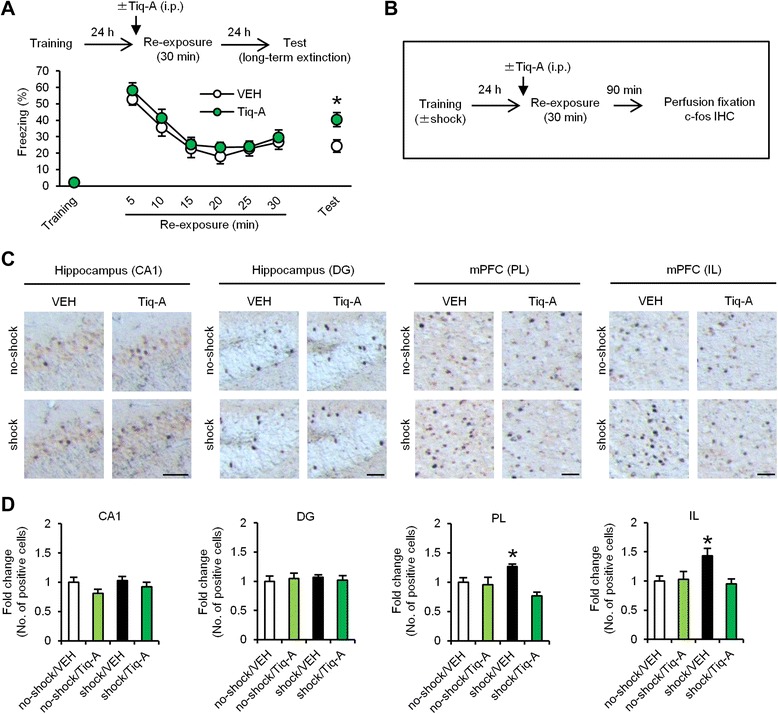
PARP-1 inactivation blocks c-fos induction in the mPFC when memory is extinguished. **a** Effects of the systemic injection of Tiq-A at 30 min before the 30 min re-exposure on long-term extinction (VEH, *n* = 17; Tiq-A, *n* = 15). *p < 0.05, compared with the VEH group at the test. **b** Experimental design for IHC. **c** Representative immunohistochemical staining of c-fos-positive cells in the CA1, DG, PL, and IL regions from the indicated mice. Scale bar, 50 μm. **d** Expression of c-fos in the CA1, DG, PL, and IL regions (*n* = 8–13 for each group). The expression of c-fos in each group is expressed as the ratio of the no-shock/VEH group to the other groups. *p < 0.05, compared with the other groups. Error bars indicate SEM

To examine whether the systemic injection of Tiq-A blocks long-term extinction, we performed a similar experiment as in Fig. 3a, except the mice were systemically injected with Tiq-A at 30 min before the 30 min re-exposure. The VEH and Tiq-A groups showed comparable within-session extinction (decrease over time, *F*_(5,150)_ = 31.728, *p* < 0.05; overall freezing, *F*_(1,30)_ = 0.691, *p* > 0.05; Fig. 6a). Two-way ANOVA revealed a significant drug × time (last 5 min in re-exposure vs. test) interaction (drug, *F*_(1,30)_ = 3.187, *p* > 0.05 ; time, *F*_(1,30)_ = 2.516, *p* > 0.05; interaction, *F*_(1,30)_ = 6.014, *p* < 0.05; Fig. 6a). The Tiq-A group froze significantly more than the VEH group during the test (*p* < 0.05; Fig. 6a). Similarly to Fig. 3, these observations indicated that PARP-1 inactivation by Tiq-A blocks long-term fear extinction.

Similarly to Fig. 5, we performed IHC at 90 min after the 30 min re-exposure using four groups. At 30 min before re-exposure, these groups were systemically injected with Tiq-A or VEH (Fig. 6b). Two-way ANOVA revealed a significant conditioning × drug interaction in the PL and IL regions of the mPFC (PL: conditioning, *F*_(1,38)_ = 0.252, *p* > 0.05; drug, *F*_(1,38)_ = 12.902, *p* < 0.05; interaction, *F*_(1,38)_ = 8.967, *p* < 0.05; IL: conditioning, *F*_(1,38)_ = 2.683, *p* > 0.05; drug, *F*_(1,38)_ = 4.277, *p* < 0.05; interaction, *F*_(1,38)_ = 5.511, *p* < 0.05; 6c and d), but not in the hippocampal CA1 and DG regions (CA1: conditioning, *F*_(1,38)_ = 0.833, *p* > 0.05; drug, *F*_(1,38)_ = 3.476, *p* > 0.05; interaction, *F*_(1,38)_ = 0.303, *p* > 0.05; DG: conditioning, *F*_(1,38)_ = 0.057, *p* > 0.05; drug, *F*_(1,38)_ = 0.001, *p* > 0.05; interaction, *F*_(1,38)_ = 0.341, *p* > 0.05; Fig. 6c and d). The shock/VEH group showed significantly more c-fos-positive cells in the PL and IL areas compared with the other groups (*p* < 0.05; Fig. 6c and d). These results indicated that PARP-1 inactivation blocks c-fos induction in the mPFC when memory is extinguished.

Collectively, our results suggested that PARP-1 activity contributes to the consolidation, reconsolidation, and extinction of contextual fear memory through the activation of new gene expression.

### Inhibition of PARP-1 activity does not affect locomotor activity or anxiety-related behavior

**Fig. 7:**
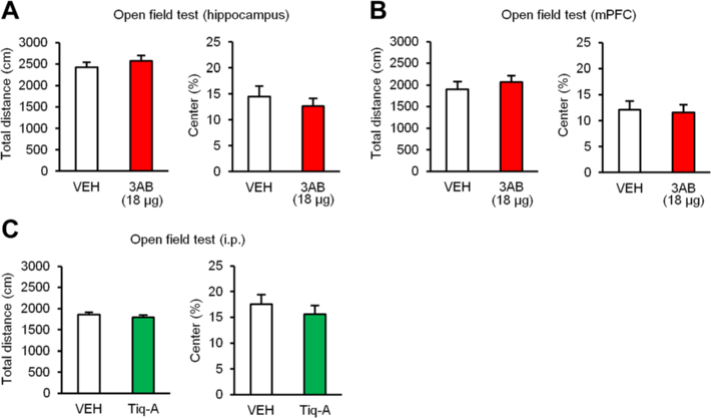
Inhibition of PARP-1 activity does not affect locomotor activity or anxiety-related behavior. **a**, **b** Effects of a micro-infusion of 3AB into the dorsal hippocampus (**a**) or mPFC (**b**) at 5 min before the open field test (dorsal hippocampus: VEH, *n* = 7; 3AB, *n* = 8; mPFC: VEH, *n* = 9; 3AB, *n* = 9). **c** Effects of a systemic injection of Tiq-A at 30 min before the open field test (VEH, *n* = 20; Tiq-A, *n* = 20). Total path length for 5 min (left panel). Percentage of time spent in the center for 5 min (right panel). Error bars indicate SEM

To test the possibility that the impairment of memory processes observed by inhibiting PARP-1 activity is attributed to abnormal locomotor and/or emotional behavior, we investigated the effects of a micro-infusion of 3AB into the dorsal hippocampus or mPFC on locomotor activity and anxiety-related behavior in an open field test. The mice received a micro-infusion of 3AB or VEH into the dorsal hippocampus (18 μg/side; Fig. 7a) or mPFC (18 μg; Fig. 7b), respectively, at 5 min before the test. One-way ANOVA revealed no significant effects of drug on total path or percentage of time spent in the center of the field (hippocampus: total distance, *F*_(1,13)_ = 0.782, *p* > 0.05; % center, *F*_(1,13)_ = 0.561, *p* > 0.05; mPFC: total distance, *F*_(1,16)_ = 0.479, *p* > 0.05; % center, *F*_(1,16)_ = 0.066, *p* > 0.05; Fig. 7a and b), indicating that a micro-infusion of 3AB into the dorsal hippocampus or mPFC does not affect locomotor activity or anxiety-like behavior. We next examined the effects of a systemic injection of Tiq-A. Similarly to the results of 3AB micro-infusion, Tiq-A treatment did not affect spontaneous behavior in the open field test (total distance, *F*_(1,38)_ = 0.731, *p* > 0.05; % center, *F*_(1,38)_ = 0.616, *p* > 0.05; Fig. 7c). These results suggest that the impairment of memory processes observed by PARP-1 inhibition is not due to nonspecific effects of the drugs on locomotor activity and/or emotional behavior.

## Discussion

Recent studies have shown that PARP-1 activity and PARP-1-mediated poly ADP-ribosylation are required for LTM formation of fear memory [47, 49]. In the present study, we examined the roles of PARP-1 in the hippocampus and mPFC in the consolidation, reconsolidation, and extinction of contextual fear memory. Inhibiting dorsal hippocampal PARP-1 activity by a local infusion of the PARP-1 inhibitor 3AB or PJ34 blocked LTM formation without affecting STM. Similarly, inhibition of PARP-1 before or after memory retrieval disrupted PR-LTM without affecting PR-STM. Conversely, inhibition of PARP-1 in the mPFC, but not hippocampus, blocked long-term extinction without affecting within-session extinction. Therefore, these observations showed that PARP-1 activity in the hippocampus is required for the consolidation and reconsolidation of contextual fear memory, whereas PARP-1 activity in the mPFC is required for its long-term extinction.

The hippocampus is known to play an essential role in the consolidation of contextual fear memory [36–38]. Previous studies have shown that PARP-1-mediated poly ADP-ribosylation occurs in the hippocampus after training in an object recognition task [47, 48]. Likewise, we also found that inhibiting hippocampal PARP-1 activity blocked the consolidation of contextual fear memory. Therefore, the induction of poly ADP-ribosylation following PARP-1 activation in the hippocampus is suggested to play a critical role in hippocampus-dependent memory consolidation.

Previous studies have shown that histone modifications, including phosphorylation, acetylation, and methylation, in the hippocampus and mPFC are necessary for the consolidation, reconsolidation, and extinction of fear memory [32, 34, 54, 55]. Indeed, these histone modifications are required for the transcriptional activation of brain-derived neurotrophic factor (BDNF) and Zif268, both of which contribute to the progress of gene expression-dependent memory processes [34, 54, 55]. Interestingly, histones, whose modification remodels chromatin structure, have been shown to be a major target of poly ADP-ribosylation mediated by PARP-1, resulting in transcriptional regulation [1, 3–6, 56]. Most importantly, previous studies have suggested that chromatin remodeling through poly ADP-ribosylation is necessary for memory consolidation [48]. In this study, we showed that inhibiting PARP-1 activity not only disrupted the consolidation, reconsolidation, and extinction of contextual fear memory but also blocked the induction of c-fos expression observed in these memory processes. Collectively, it is likely that the PARP-1-mediated poly ADP-ribosylation of histones and subsequent activation of gene expression are required for the consolidation, reconsolidation, and extinction of contextual fear memory. Therefore, it is important to identify critical target proteins that are poly ADP-ribosylated by PARP-1 and required for gene expression-dependent memory processes.

A previous study showed that the systemic injection of 3AB did not affect locomotor activity in an open field test [57]. Likewise, the present study also indicated that the systemic injection of Tiq-A and local infusion of 3AB into the dorsal hippocampus or mPFC did not affect locomotor activity or anxiety-related behavior. Therefore, these observations suggested that the memory impairment observed following the infusion of PARP-1 inhibitors is not due to abnormalities of spontaneous locomotor activity or anxiety-related behavior, but rather appears to be due to the inhibition of memory processes.

LTM formation in *Aplysia* is inhibited by 3AB administration prior to operant conditioning, but not immediately after training [45]. Likewise, the present study showed that LTM formation of contextual fear was disrupted by the infusion of PARP-1 inhibitors into the dorsal hippocampus at 5 min before, but not immediately after, training. Our previous study indicated that inhibition of gene expression by the administration of the protein synthesis inhibitor anisomycin immediately after training disrupted LTM of contextual fear [41]. These findings suggested that PARP-1 activation is likely to be necessary in the early stage of memory consolidation processes such as during or immediately after training.

The molecular mechanisms of both memory consolidation and reconsolidation seem to be similar, but also show differences from each other [58, 59]. For example, hippocampal BDNF is required for the consolidation of contextual fear memory, while hippocampal Zif268 is required for the reconsolidation of this memory [37, 60, 61]. Unlike in the case of the infusion of PARP-1 inhibitors against the consolidation of contextual fear memory, the present study showed that both the pre- and post-re-exposure local infusion of PARP-1 inhibitors into the dorsal hippocampus blocked memory reconsolidation. Therefore, this result suggests that the consolidation and reconsolidation mechanisms are similar, but they work differently; that is, PARP-1 activation functions in different time windows during memory consolidation and reconsolidation.

Transcriptional activation by the transcription factor cAMP responsive element binding protein (CREB) is known to be necessary for the consolidation, reconsolidation, and long-term extinction of contextual fear memory [10, 14, 36, 39, 62]. A recent study showed that PARP-1 activity is necessary to induce hippocampal expression of the CREB-target gene c-fos after training in an object recognition task [48]. Another study showed that PARP-1 inhibition in the hippocampus leads to decreases in cAMP level, PKA activity, PKAcα expression, and CREB phosphorylation. Thus, these findings suggest that PARP-1 positively regulates the cAMP-PKA-CREB pathway [49]. In this study, we found that PARP-1 activation is required for memory consolidation, reconsolidation, and long-term extinction; furthermore, PARP-1 activity is required for c-fos induction when memory is consolidated, reconsolidated, and extinguished. Taken together, these findings raise the possibility that PARP-1 facilitates memory processes through the activation of CREB-mediated gene expression.

## Conclusions

In the current study, we showed that PARP-1 activity in the hippocampus is necessary for both the consolidation and reconsolidation of contextual fear memory; moreover, this activity in the mPFC is necessary for long-term extinction. Taken together, our study suggests that PARP-1-mediated poly ADP-ribosylation has a crucial role not only in memory formation of contextual fear conditioning but also in regulation after memory retrieval.

## Methods

### Mice

All experiments were conducted according to the *Guide for the Care and Use of Laboratory Animals, Japan Neuroscience Society and Tokyo University of Agriculture*. All animal experiments performed in this study were approved by the Animal Care and Use Committee of Tokyo University of Agriculture. Male C57BL/6N mice were obtained from Charles River (Yokohama, Japan). The mice were housed in cages of 5 or 6, maintained on a 12 h light/dark cycle, and allowed *ad libitum* access to food and water. The mice were at least 8 weeks of age at the start of the experiments, and all behavioral procedures were conducted during the light phase of the cycle. All experiments were conducted blind to the treatment condition of the mice.

### Surgery and micro-infusion

**Fig. 8:**
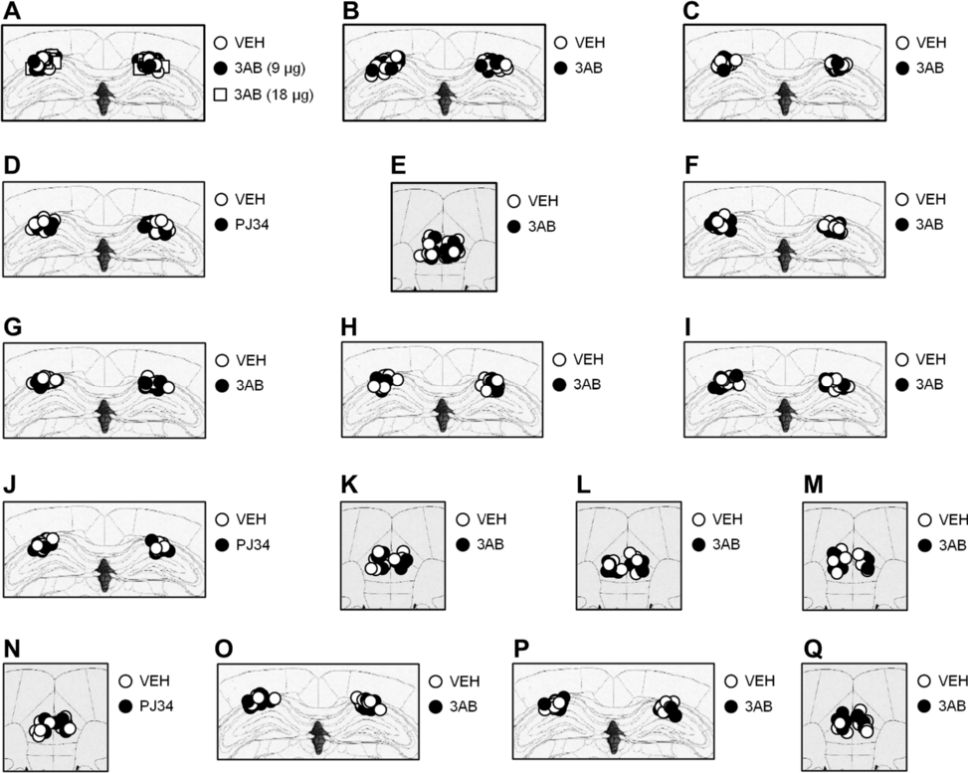
Cannula tip placements in the dorsal hippocampus and mPFC. Cannula tip placements from mice infused with each drug shown in Fig. 1A (**a**), Fig. 1B (**b**), Fig. 1C (**c**), Fig. 1D (**d**), Fig. 1E (**e**), Fig. 2A (**f**), Fig. 2B (**g**), Fig. 2C (**h**), Fig. 2D (**i**), Fig. 2E (**j**), Fig. 2F (**k**), Fig. 3A (**l**), Fig. 3B (**m**), Fig. 3C (**n**), Fig. 3D (**o**), Fig. 7A (**p**), and Fig. 7B (**q**). Schematic drawing of coronal sections from all micro-infused animals (dorsal hippocampus, 1.94 mm posterior to the bregma; mPFC, 1.94 mm anterior to the bregma) [68]. Only mice with needle tips within the boundaries of the dorsal hippocampus or mPFC were included in the data analysis

Surgery was performed as described previously [39–41, 63–67]. Under Nembutal anesthesia and using standard stereotaxic procedures, a stainless steel guide cannula (22 gauge) was implanted into the dorsal hippocampus (−1.9 mm, ± 1.8 mm, −1.9 mm) or mPFC (2.0 mm, ± 0 mm, −1.6 mm) [68]. The mice were allowed to recover for at least 1 week after surgery. Infusions into the dorsal hippocampus (0.5 μL/side) or mPFC (0.5 μL) were made at a rate of 0.25 μL/min. The injection cannula was left in place for 2 min after infusion. Only mice with a cannulation tip within the boundaries of the dorsal hippocampus or mPFC were included in the data analysis. Cannulation tip placements are shown in Fig. 8.

### Drugs

The PARP-1 inhibitors 3AB (18 or 36 μg/μL; Sigma, MO, USA), PJ34 (0.2 mM; Enzo Life Sciences, NY, USA), and Tiq-A (0.5 mg/kg; Sigma) were dissolved in dimethyl sulfoxide (DMSO). Local administration of PJ34 (0.2 mM) into the lateral ventricle blocked PARP-1 activation and the consolidation of object recognition and inhibitory avoidance memories [47]. Systemic administration of Tiq-A (0.5 mg/kg) blocked PARP-1 activation and the consolidation of object recognition memory [48]. 3AB and PJ34 were diluted in artificial cerebrospinal fluid and saline, respectively, to a final DMSO concentration of 10 %. Tiq-A was diluted in saline to a final DMSO concentration of 1 %.

### Contextual fear conditioning task

The mice were handled for 5 consecutive days prior to the commencement of contextual fear conditioning. The mice were trained and tested in conditioning chambers (17.5 × 17.5 × 15 cm) that had a stainless steel grid floor through which a footshock could be delivered [25, 39, 41, 63, 69]. Training consisted of placing the mice in the chamber and delivering an unsignaled footshock (2 s duration, 0.4 mA) at 148 s later, and then the mice were returned to their homecage at 30 s after the footshock (training).

For the experiments to examine the effects of drug treatment on memory consolidation, the mice received a micro-infusion of 3AB, PJ34, or VEH into the dorsal hippocampus or mPFC at 5 min before or immediately after training (see Fig. 1). At 2 or 24 h after training, the mice were placed back in the training context for 5 min (test). For the experiments to examine the effects of drug treatment on memory reconsolidation or extinction, the mice were trained and placed back in the training context at 24 h later for 0, 3, or 30 min (re-exposure). The mice received a micro-infusion of 3AB, PJ34, or VEH into the dorsal hippocampus or mPFC at 5 min before or immediately after re-exposure (see Figs. 2 and 3). At 2 or 24 h after re-exposure, the mice were once again placed back in the training context for 5 min (test). In the case of 0 min re-exposure, the mice remained in their homecage (not re-exposed to the training chamber), but were treated with the drugs. Memory was assessed as the percentage of time spent freezing in the training context. Freezing behavior (defined as complete lack of movement, except for respiration) was measured automatically as described previously (O’HARA & CO., LTD., Tokyo, Japan) [70]. Tiq-A or VEH was systemically injected (an i.p. injection) at 30 min before training or re-exposure (see Figs. 4, 5, and 6).

### Immunohistochemistry

IHC was performed as described previously [39, 41, 63, 65, 67]. After anesthetization, all mice were perfused with 4 % paraformaldehyde. Brains were then removed, fixed overnight, transferred to 30 % sucrose, and stored at 4 °C. Coronal sections (30 μm) were cut in a cryostat. The sections were pretreated with 4 % paraformaldehyde for 20 min and 3 % H_2_O_2_ in methanol for 1 h, followed by incubation in a blocking solution (phosphate-buffered saline [PBS] plus 1 % goat serum albumin, 1 mg/mL bovine serum albumin, and 0.05 % Triton X-100) for 3 h at 4 °C. Consecutive sections were incubated with a polyclonal rabbit primary antibody for anti-c-fos (Ab-5; 1:5000; Millipore, MA, USA) in the blocking solution over 2 nights at 4 °C. Subsequently, the sections were washed with PBS and incubated for 4 h at room temperature with biotinylated goat anti-rabbit IgG (SAB-PO Kit; Nichirei Biosciences, Tokyo, Japan), followed by 1 h at room temperature in streptavidin-biotin-peroxidase complex (SAB-PO Kit). Immunoreactivity was detected with a DAB substrate kit (Nichirei Biosciences). Structures were defined anatomically according to the atlas of Paxinos and Franklin [68]. Quantification of c-fos-positive cells in sections (100 × 100 μm) of the mPFC (bregma between +2.10 and +1.98 mm) and dorsal hippocampus (bregma between −1.46 and −1.82 mm) was performed using a computerized image analysis system (WinROOF version 5.6 software; Mitani Corporation, Fukui, Japan). Immunoreactive cells were counted bilaterally with a fixed sample window across at least 3 sections by an experimenter blind to the treatment condition. The expression levels of c-fos in each group were expressed as the ratio of the averaged values in the no-shock/VEH control group.

### Open field test

An open field test was performed as described previously [71]. The mice received a micro-infusion of 3AB or VEH into the dorsal hippocampus or mPFC at 5 min before the test (see Fig. 7a and b). Tiq-A or VEH was systemically injected (an i.p. injection) at 30 min before the test (see Fig. 7c). The mice were placed into the center of a square open field chamber (50 × 50 × 40 cm) that was surrounded by white walls. The total length of the path traveled (total distance) and the time spent in the center square (30 × 30 cm; % center) were measured over the course of 5 min using an automatic monitoring system (O’HARA & CO., LTD.).

### Data analysis

One-way or two-way factorial or repeated ANOVA followed by *post hoc* Newman-Keuls comparison were used to analyze the effects of drug, time, and conditioning. A paired t-test was used to analyze the differences in freezing within each group between two phases (first 5 min in 30 min re-exposure vs. test). All values in the text and figure legends represent the mean ± standard error of the mean (SEM).
